# A Case of Breast Cancer Metastasizing to the Ampulla of Vater

**DOI:** 10.7759/cureus.58396

**Published:** 2024-04-16

**Authors:** Matthew Tjahja, Phi P Tran, Paula Danika Binsol, Jonathan C Ramirez

**Affiliations:** 1 Internal Medicine, Baylor Scott and White Temple Medical Center, Temple, USA; 2 Pathology, Baylor Scott and White Temple Medical Center, Temple, USA; 3 Gastroenterology, Baylor Scott and White Temple Medical Center, Temple, USA

**Keywords:** egd and eus, immunohistochemical study, palliative treatment, ampulla of vater, breast cancer metastasis

## Abstract

Secondary tumors of the ampulla of Vater are exceedingly rare and associated with relatively poor prognosis. Tumors of the ampulla are classified into four distinct subtypes based on the location and involvement of surrounding structures. Most reported cases are of renal cell or malignant skin melanoma primary with only five previously reported cases of breast primary found in a literature review.

We present a 72-year-old woman with metastatic breast cancer to the ampulla of Vater as well as multiple bones. She had a history of breast cancer status post bilateral mastectomy and chemo 27 years prior. She presented to the hospital with altered mental status and was found to have an acute liver injury. Magnetic resonance cholangiopancreatography revealed a distended gallbladder and an indeterminate left retroperitoneal mass concerning for cystic or necrotic lymphadenopathy. Endoscopy then showed an edematous and erythematous periampullary region, which was biopsied and returned positive for carcinoma. Immunohistochemical staining of the retroperitoneal mass returned positive for keratin, estrogen receptor, GATA3, and MOC31 and negative for progesterone receptor, WT1, calretinin, and E-cadherin. The periampullary region's immunohistochemistry returned positive for pankeratin (AE1/AE3) and CD138 and negative for CD45 and S100, supporting a diagnosis of primary breast carcinoma.

The average time from diagnosis of breast cancer to metastasis was found to be 2.5 years. Endoscopic visual presentation of metastatic cancer to the ampulla is indistinguishable from that of primary cancers. Thus, a biopsy with cytology and immunohistochemical analysis is necessary for diagnosis.

Management of secondary ampullary tumors requires a multidisciplinary team, including gastroenterology, surgery, oncology, and often palliative care. Secondary tumors have been found to be treated by any combination of Whipple's resections, chemotherapy, drainage/stenting, and endoscopic ampullectomy.

## Introduction

The ampulla of Vater, discovered by Abraham Vater in 1720, consists of portions of the duodenum, the terminal tract of the pancreatic duct, and the last portion of the common bile duct [1]. Primary tumors of the ampulla have been classified into four distinct subtypes based on location: (1) intra-ampullary, comprising 25% of cases and limited to intra-ampullary papillary-tubular neoplasms with zero to minimal duodenal surface involvement, (2) ampullary-ductal, comprising 15% of cases and involving thickening of the common bile duct and/or pancreatic duct, (3) peri-ampullary-duodenal, comprising 5% and described as “massive exophytic, ulcero-fungating tumors growing into the duodenal lumen with only minimal intra-ampullary luminal involvement”, and (4) ampullary carcinoma-not otherwise specified, comprising 55% of cases and has characteristics that were non-specific to the above 3 subtypes [2].

Among secondary malignancies of the upper GI tract, lung cancer, breast cancer, and malignant melanoma have been reported to be the most common [3]. Secondary malignancies of the ampulla have also been reported albeit much more rarely. One literature review revealed 32 cases of metastatic cancer to the ampulla of Vater from 1989-2017, further showing the rarity of this event. Of these, 9 were primarily malignant melanoma of the skin, 11 were primary clear cell carcinoma of the kidney, and only 4 were breast carcinoma primary [4]. An additional two cases have since been reported-one breast primary, and one renal primary [5].

In this case report, we present a 72-year-old patient who was found to have ampullary metastasis from breast carcinoma despite treatment with radical mastectomy 27 years prior as well as chemotherapy, which was stopped in 2000.

## Case presentation

A 72-year-old female with a history of breast cancer status post bilateral mastectomy and chemotherapy, hypertension, hyperlipidemia, and osteoarthritis presented to the hospital with altered mental status and was found to have elevated liver function tests with a total bilirubin of 4.0, alkaline phosphatase (ALP) of 787, aspartate transaminase (AST) of 1454, alanine transaminase (ALT) of 1342, and ammonia of 61. Abdominal computed tomography (CT) was concerning for possibly thrombosed celiac artery aneurysm, multiple liver lesions of unknown etiology, and a mildly distended gallbladder with a common bile duct measuring 8 mm but no stones. Magnetic resonance cholangiopancreatography again demonstrated a distended gallbladder with mild wall thickening and pericholecystic fluid and no evidence of obstructing stones. It also demonstrated an indeterminate left retroperitoneal mass concerning for cystic or necrotic lymphadenopathy.

**Figure 1:**
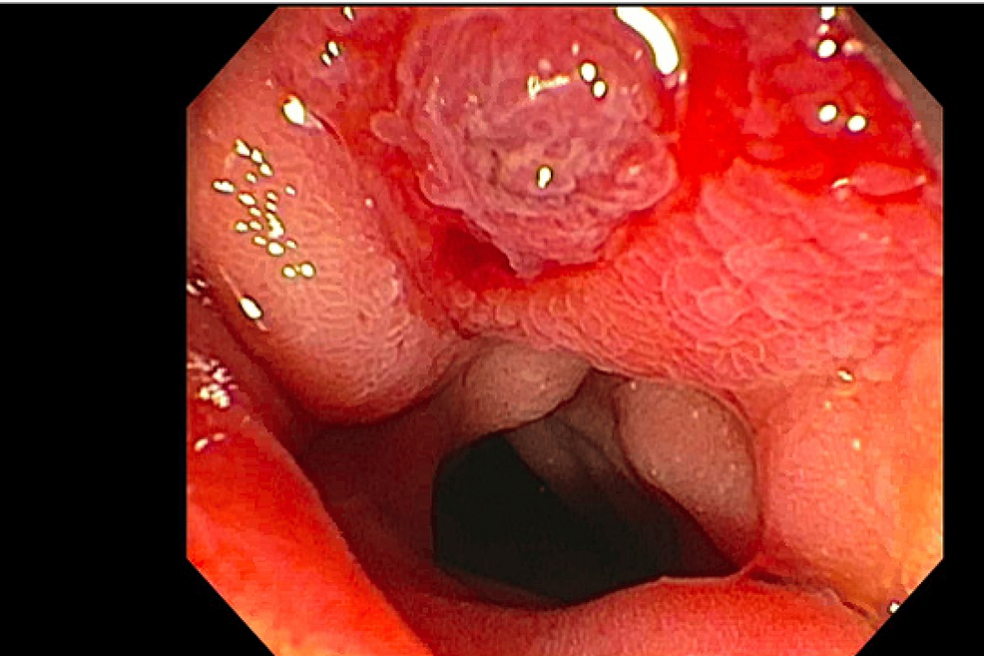
Erythematous and edematous periampullary lesion

**Figure 2:**
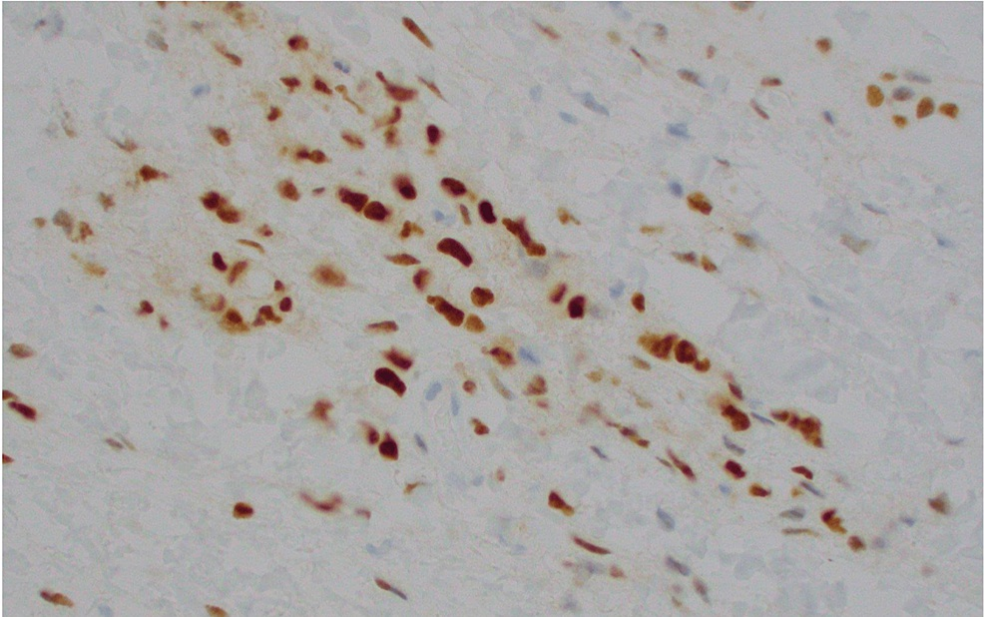
Retroperitoneal mass cells positive for ER (estrogen receptor) on immunohistochemical staining

**Figure 3:**
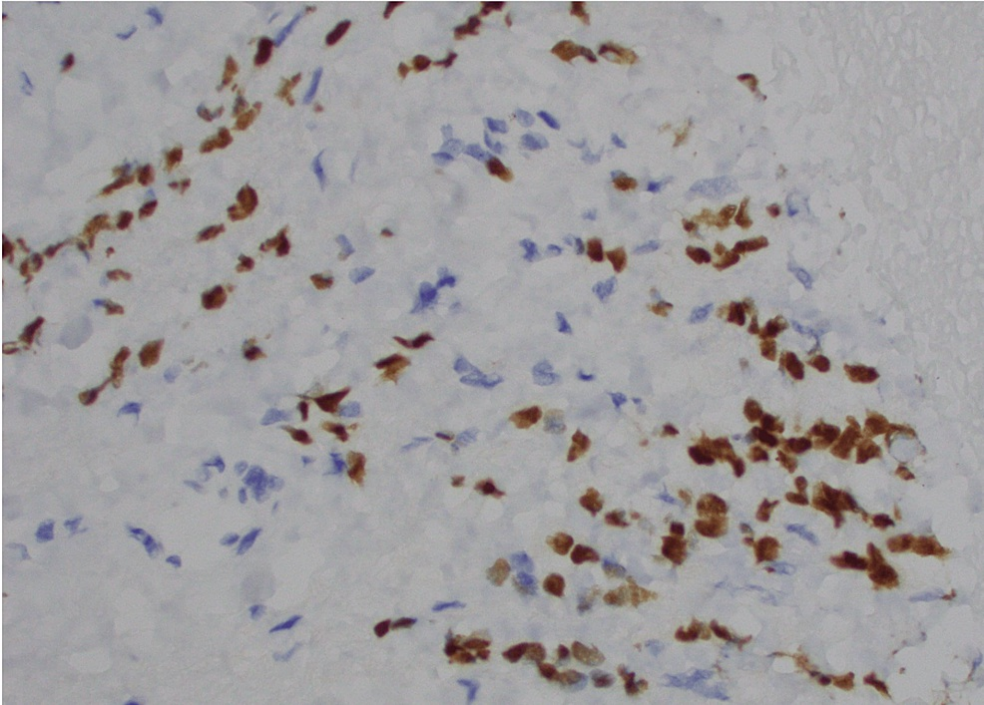
Retroperitoneal mass cells positive for GATA3 on immunohistochemical staining

**Figure 4:**
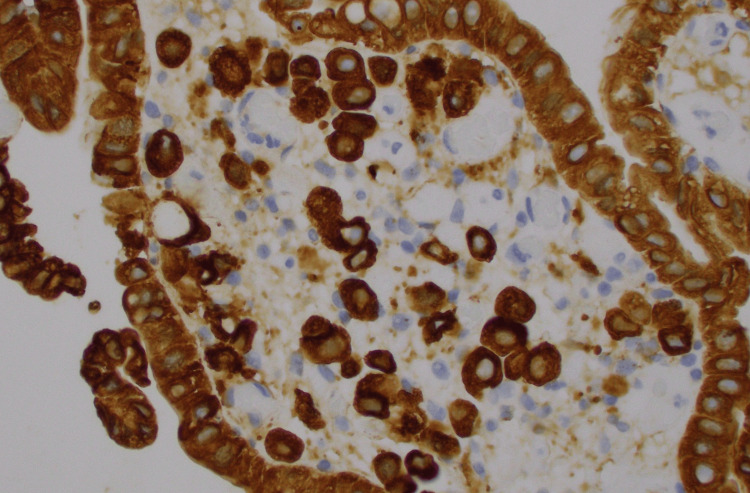
Periampullary mass cells positive for pankeratin on immunohistochemical staining

The patient underwent esophagogastroduodenoscopy and endoscopic ultrasound, which revealed an edematous and erythematous periampullary region (Figure 1), moderate non-erosive gastritis, as well as a left retroperitoneal mass. Immunohistochemical analysis of the left retroperitoneal mass revealed atypical cells positive for keratin, estrogen receptor (Figure 2), GATA3 (Figure 3), Moc31 (weak), and negative for progesterone receptor, WT1, and calretinin and E-cadherin. Pathology of the periampullary region revealed intestinal mucosa focally involved by carcinoma that was positive for pankeratin (AE1/AE3) (Figure 4) and CD138, and negative for CD45 and S100. These atypical cells were morphologically similar to those in the retroperitoneal mass, supporting the diagnosis of metastatic carcinoma of the breast primary.

A whole-body positron emission tomography scan revealed multiple focal areas of nonspecific increased tracer activity including multiple ribs, multiple vertebrae, and multiple bones within her extremities. Due to the extent of metastatic disease and lack of persistent cholestasis, an ampullectomy/sphincterotomy was not performed. She has been treated with palliative chemotherapy with palbociclib, letrozole, and zoledronic acid. She continues to undergo regular CT scans and bloodwork with no overt evidence of disease progression to date.

## Discussion

Metastasis of any type to the ampulla of Vater can present as upper gastrointestinal bleeding, obstructive jaundice, nausea, and vomiting. In our case, the patient initially presented with altered mental status and was found to have elevated liver function tests.

For the five cases of breast carcinoma with metastasis to the ampulla of Vater described by Sarrochi et al. and Ali et al., the average time from diagnosis of breast cancer to metastasis was roughly 2.5 years [4,5]. In our case, our patient’s initial diagnosis of breast cancer was roughly 27 years prior. However, it should be noted that roughly four years ago, the patient was found to have a mass on a mammogram, which was subsequently removed. Thus, the argument could be made that this metastasis was roughly four years from a second diagnosis of breast cancer.

The endoscopic presentation of metastatic cancer to the ampulla is indistinguishable from that of primary cancers. Most lesions will present as polypoid, irregular, and friable masses, much like that of our patient. Definitive diagnosis thus requires biopsy with cytology and immunohistochemical analysis [6].

Due to the high tumor burden and presence of metastasis to multiple bones in our patient, curative treatment was not pursued. Primary adenocarcinoma of the ampulla is most frequently treated with pancreaticoduodenectomy, particularly when the metastatic burden of the disease is low [7]. There is limited data on the treatment of secondary tumors but, upon review of the literature, it appears that secondary tumors of the ampulla have been treated with Whipple’s resections, chemotherapy (both with surgery and alone), drainage/stenting, and endoscopic ampullectomy. In a review of the treatment of benign ampullary disease, a surgical approach was found to have increased length of stay, morbidity, and readmission rates suggesting that if the disease is localized, an endoscopic approach should be considered [8]. However, endoscopic ampullectomy also confers an increased risk of pancreatitis, bleeding, and perforation [9]. Other factors to consider during surgical and treatment planning include lymphatic nodal and vessel involvement [10].

## Conclusions

Metastatic cancer to the ampulla of Vater is a rare occurrence that most frequently occurs with renal cell carcinoma and malignant skin melanoma primaries. However, breast cancer metastasis to the ampulla has also been reported. Initial presenting symptoms can include upper gastrointestinal bleeding, jaundice, nausea, and vomiting. A biopsy with cytology and immunohistochemistry is necessary to distinguish primary vs. secondary tumors. Treatment should be individualized based on the patient’s metastatic burden but is usually palliative due to the poor prognosis and can include any combination of surgery, chemotherapy, and ampullectomy.
